# Predicting Compliance with Sanitary Behaviors among Students in Higher Education During the Second COVID-19 Wave: The Role of Health Anxiety and Risk Perception

**DOI:** 10.5334/pb.1171

**Published:** 2023-01-05

**Authors:** Sarah Dekeyser, Emilie Schmits, Fabienne Glowacz, Olivier Klein, Mathias Schmitz, Robin Wollast, Vincent Yzerbyt, Olivier Luminet

**Affiliations:** 1 Research Institute for Psychological Sciences, UCLouvain, Louvain-la-Neuve, Belgium; 2 Psychologie Clinique de la Délinquance, Unité de Recherche Adaptation, Résilience et Changement, Université de Liège, Liège, Belgium; 3 Center for Social and Cultural Psychology, Université Libre de Bruxelles, Brussels, Belgium; 4 Fund for Scientific Research (FRS-FNRS), Brussels, Belgium

**Keywords:** health anxiety, risk perception, sanitary behaviors, students, COVID-19

## Abstract

To limit the spread of COVID-19, public authorities have recommended sanitary behaviors such as handwashing, mask-wearing, physical distancing, and social distancing. We recruited a large sample of higher education students in Belgium (*N* = 3201–3441) to investigate the role of sociodemographic variables, mental health, previous COVID-19 infections, academic involvement, and risk perception on adherence to these sanitary behaviors. This cross-sectional study took place during the second COVID-19 wave in Belgium, between February and March 2021. Analyses showed that living alone, being female, later in the academic curriculum, having higher general and health anxiety, higher academic involvement, and higher risk perception were positively associated with adherence to the four aforementioned sanitary behaviors. Conversely, previous infection with COVID-19 and having been quarantined were negative predictors. Our results show a set of predictors highly similar for the four sanitary behaviors. We discuss potential initiatives to increase adherence to sanitary behaviors in this group of highly educated youngsters.

The emergence of the COVID-19 pandemic has dramatically changed individuals’ daily behaviors. In the hope of limiting the propagation of the virus, the World Health Organization promptly published a list of recommended sanitary behaviors, such as washing hands, wearing a face mask, physically distancing oneself from others, and avoiding social gatherings (World Health Organization, 2020). The primary goal of this cross-sectional study is to identify predictors of college students’ adherence to these four central sanitary behaviors that limit the spreading of COVID-19. This study took place during a moment of high infection rate and strict measures imposed by public authorities. We investigated a series of key variables previously associated with the adoption of sanitary behaviors among the general population. We also measured these four behaviors with new scales.

Recent studies conducted during the COVID-19 pandemic on the college student population showed that 50 percent of them never wash their hands after coughing, sneezing, or blowing their nose (Cohen et al., 2020), but most of them tend to wear face masks on a regular basis (Cohen et al., 2021; DeJonckheere et al., 2021). However, these studies focused on American samples, and there are variations in compliance among college students across countries. For instance, compared to their American counterparts (Cohen et al., 2020), most of the college students in Sweden or in Lebanon tend to comply with preventive behaviors recommended by their government (Berman et al., 2022; Elhadi et al., 2020). Yet, because of their strong need for and active involvement in social connections compared to other age groups (Wrzus et al., 2013), young adults from 18 to 25 years old are a crucial population to target for health prevention. They are likely to be at a higher risk for facilitating the spread of any given virus, but concurrently, sustained social interactions between young adults are needed for their mental health (Milner et al., 2015), especially during the COVID-19 pandemic (Okabe-Miyamoto & Lyubomirsky, 2021). The COVID pandemic, therefore, represents a dilemma for a population that needs to balance the risks of spreading the virus while requiring frequent and sustained social contacts.

We focused on tertiary-level students for several reasons besides the amount of their social connections. The outbreak had significant consequences for college students, particularly with regards to their mental health (Essadek & Rabeyron, 2020; Glowacz & Schmits, 2020; Li, Wang, et al., 2021). During the pandemic, the rates of depression and anxiety symptoms increased in the college student population all around the world (Alam et al., 2021; Chouksey & Agrawal, 2021; Li, Zhao, et al., 2021; Schmits et al., 2021; Sheldon et al., 2021; Wieczorek et al., 2021), regardless of the gender, the field or the year of education (Li, Wang, et al., 2021). They also experienced high concerns regarding their future and insecurities arising from the COVID-19 situation (Alam et al., 2021; Gupta & Agrawal, 2021). As higher education institutions across the world had to suddenly close their doors, their education was also largely impacted, with classes and exams shifted to online modalities (Marinoni et al., 2020; Pokhrel & Chhetri, 2021; Tarkar, 2020). This closure and its related changes were coupled with limitations regarding accessibility for many college students (Rashid & Yadav, 2020), leading to a time of high uncertainty.

Furthermore, tertiary-level students are often viewed as scapegoats of the pandemic due to the intensity of their social activities (Danon et al., 2013) and their involvement in rule-breaking social events (e.g., lockdown parties). Surprisingly, this population has been overlooked in current studies on the adherence to sanitary behaviors. In addition, current studies that have considered college students had limited sample sizes, ranging from 255 (e.g., Varol et al., 2021) to 1015 (e.g., Park et al., 2020) participants. Given this lacuna, the present study aims to shed light on the determinants of these sanitary behaviors by including a much larger number of participants. This study took place during a sensitive time (March 2021) for higher education students in Belgium. Indeed, a partial lockdown started in October 2020, and all courses went online for the second time that year. Also, with the infection rate in Belgium rapidly rising yet again in March 2021 (Sciensano, 2021c), students could not return to campus, preventing them from having clear perspectives on their return to campus.

## General and Health Anxiety

Research on the general population suggests several dimensions that can predict adherence to sanitary behaviors. First, general anxiety has been highlighted as a determinant of behavioral compliance, although not systematically. Whereas some studies found a positive relationship with adherence to sanitary behaviors (Oral & Gunlu, 2021), others did not (Mevorach et al., 2021). Recent research found that excessive worrying is a key-symptom in anxiety during the COVID-19 pandemic (Heeren et al., 2021), but only a few previous studies have focused on the specific anxiety related to the health. Health anxiety refers to the preoccupation of having a serious disease (Johnstone et al., 2010), and is associated with higher anxiety related to the COVID-19 (Jungmann & Witthöft, 2020; Seyed Hashemi et al., 2020). Furthermore, in their study on determinants of handwashing and social distancing, Bigot and colleagues (2021) found that higher health anxiety predicts more handwashing. Therefore, trait health anxiety might play a key-role in compliance with sanitary behaviors during times of pandemic (Asmundson & Taylor, 2020; Sauer et al., 2020).

## Risk perception and past contamination

Risk perception and experiences with the COVID-19, via infection or quarantine, are other variables associated with adherence to sanitary behaviors within the general population (Schmälzle et al., 2017). Risk perception related to the COVID-19 refers to one’s evaluations of hazard they might be exposed to the virus and its undesirable effects (Cori et al., 2020). Risk perception involves two aspects: the perceived probability of being infected, and the perceived risk of developing severe symptoms (Loewenstein et al., 2001; Wolff et al., 2019). Risk perception has already been reported as a factor of compliance with preventive measures during previous pandemics, such as the H1N1 pandemic (Gilles et al., 2011; Wheaton et al., 2012). During the COVID-19 pandemic, higher risk perception related to the coronavirus has been associated with higher compliance with sanitary behaviors in different countries, such as China, United Kingdom or United-States (Bowman et al., 2021; de Bruin & Bennett, 2020). Conversely, individuals who had been infected with COVID-19 seem to be less likely to follow the recommended behaviors (Pascaru, 2021), probably because they feel less at risk of future infection.

## Academic motivation

Previous research found that adoption of COVID-19-related sanitary behaviors were related to prosocial motivation (Ai et al., 2021; Jordan et al., 2021). However, these sanitary behaviors were also related to individuals’ autonomous motivation (Morbée et al., 2021; Motivation barometer, 2021a, 2021b, 2021c, 2021d, 2021e, 2021f; Schmitz et al., 2021). Within the specific college student’s population, autonomous motivation is closely related to academic motivation (Karimi & Sotoodeh, 2020). As Belgian college students were not allowed on campus, and outdoor activities were prohibited, they mostly stayed at home (Wismans et al., 2020), which negatively influenced their academic involvement (Meeter et al., 2020). Hence, this unpleasant situation of social isolation, and the decreased motivation could lead to a lack of compliance with sanitary behaviors, especially those involving an interpersonal component, namely mask wearing, physical and social distancing. Indeed, because of the lack of social connections and the prohibition to be outside, sanitary behaviors with an interpersonal dimension might be perceived as less relevant.

## Originality of the study

The present study is innovative as it measures sanitary behaviors through a set of items representing different contexts, and different ease of behaviors application (Schmitz et al., 2022; Wollast et al., 2021). Such an approach provides a higher ecological validity in comparison to studies that adopt a single overall measure for each behavior (Brouard et al., 2020; Clark et al., 2020; Hung et al., 2021; Patil et al., 2021; Wollast, Sadeghi-Bahmani, et al., 2022). Moreover, these different indices allow to identify specific determinants for each preventive behavior (i.e., handwashing, mask wearing, social distancing and physical distancing), which is more accurate than with a single measure of compliance with sanitary measures (Wismans et al., 2020).

## Method

### Participants

A total of 22,224 student participants from French-speaking tertiary-level education in Belgium completed our online survey. All institutions of higher education in the French part of Belgium were represented. This sample corresponds to approximatively 10% of the students enrolled in higher education in the French-speaking region of Belgium. As we focused on students between 18 and 25 years old, all participants older than 25 were removed from the analyses. Then, we identified multivariate outliers using the Mahalanobis distance, yielding a final sample of 20,048 valid participants (*M* = 20.89, *SD* = 1.95). Regarding their institution, 58% of students were enrolled at universities, 37% in professional colleges, and 3% in art schools. Most of the sample identified themselves as women (69.2%, for 29.8% men and 0.9% non-binary gender). Concerning their education level, 76% were enrolled in bachelor-level courses, 23% in master-level courses, and 1% in post-master-level courses (Table 1). There is a slight over-representation of students in universities, and a slight under-representation of students in professional colleges compared to the actual representation of students in higher education in Belgium (Académie de Recherche et de l’Enseignement Supérieur, 2016). Note that a paper focusing on the psychological distress of these same respondents has been published elsewhere (Schmits et al., 2021).

**Table 1:** Comparison of frequencies of students enrolled in higher education by institutions (study and total Belgian French-speaking higher education students).


INSTITUTIONS	PERCENTAGE IN THE STUDY SAMPLE	PERCENTAGE IN THE TOTAL BELGIAN FRENCH-SPEAKING HIGHER EDUCATION STUDENTS

Universities	58.5	50.6

Professional colleges	37.6	45.9

Art schools	3.9	3.5


### Measures

#### Sanitary Behaviors

We measured four sanitary behaviors with items on 5-point Likert-type scales. We used the items from a study also predicting sanitary behaviors in the context of the COVID-19 pandemic (Wollast, Schmitz, et al., 2022). The items varied in the level of difficulty of the targeted sanitary behaviors and their ease of application, with some being more difficult to follow than others. We conducted principal component analyses (Appendix A), leading to four variables, each composed of five to seven items. Items are detailed in Appendix B. All items refer to sanitary behaviors related to the context of the COVID-19 pandemic.

**Handwashing.** Participants indicated to what extent they followed the handwashing behavior (or using hand sanitizer) in six different contexts, namely a) after blowing their nose, coughing or sneezing; b) before eating; c) before and after being in a public space; d) before and after being in a private space; e) before and after touching an object frequently touched by other people; and f) at regular intervals during the day. We computed a mean index with the six items. Cronbach’s alpha was good (*α* = .80).

**Mask wearing.** Participants indicated to what extent they wore a face mask in the following situations: a) with visitors who came to their home; b) with friends; c) with family members who did not live in the same house; d) with strangers in a poorly ventilated room; and e) when they were outside where masks were not required but where the recommended physical distance (1.5 meters) could not be maintained. We computed a mean index with the five items. Cronbach’s alpha was good (*α* = .83).

**Physical distancing.** Participants indicated to what extent they followed the behaviors of physical distancing (i.e., keeping a distance of 1.5 meters from others) in the different contexts listed: a) with other people, even with friends and family members; b) with individuals who did not live in the same house; c) with others when they were outside; d) avoiding public transport or crowded spaces; and e) working/studying as much as possible at home. We computed a mean index with the five items. Cronbach’s alpha was good (*α* = .77).

**Social distancing.** Participants indicated to what extent they limited social contacts in different contexts, namely avoiding a) seeing their family outside of their house; b) seeing their friends; c) social contacts with people who do not live with them; d) small gatherings; e) gatherings in public space; f) gatherings in private spaces; and g) crowded spaces. We computed a mean index with the seven items. Cronbach’s alpha was good (*α* = .81).

#### Sociodemographic Background

We collected students’ gender (male, female, or non-binary), age, and measured socioeconomic status using the highest degree obtained by the mother or equivalent on a scale ranging from 1 “no diploma” to 8 “PhD degree”.

#### General Anxiety

The Hospital Anxiety and Depression Scale (Bocéréan & Dupret, 2014; Zigmond & Snaith, 1983) evaluated the level of anxiety and depression on a 14-item scale. We only used the anxiety subscale, composed of seven items that were used to measure anxiety symptoms (*α* = .79). The 4-point Likert-type scale ranged from “never” (=0) to “most of the time” (=3).

#### Health Anxiety

We selected three items from the Whiteley Index (Pilowsky, 1967), relying on a 5-point Likert-type scale (from 1 = totally disagree to 5 = strongly agree). Participants reported to what extent they were worried about their global health, about being sick, and to what extent they had difficulties believing their doctor when they told them that they have nothing to worry about. Cronbach’s alpha computed with the three items was not satisfactory (*α* = .61). Thus, to ensure the reliability of the measure, we selected the two out of the three items with the strongest correlation to compute a mean index of health anxiety. We kept the item on worries about global health, and the one on worries about being sick because of their moderate correlation (*r* = .51).

#### Social Support Satisfaction

The social support satisfaction index is a mean score of four items evaluating participants’ feelings of satisfaction about their relationships with family members, friends, other students, and teachers (*α* = .67).

#### Living alone

Participants indicated if they were living with other people (1) or alone (2).

#### Academic Motivation

We computed an academic motivation index with eight items using a 5-point Likert-type scale. We created a first reversed item assessed the feeling of disengagement during the last month (“*During the last month, how would you describe your investment in your study?*”). Seven other items were adapted from the Academic Motivation Scale (Vallerand et al., 1989). Among these seven items, two focused on the pleasure of studying, two on the intrinsic motivation for their academic involvement, and three on reasons for a reduction in their academic involvement. The overall Cronbach’s alpha was good (*α* = .76).

#### Experiences with the COVID-19

We asked students two questions regarding their past experiences with the COVID-19. First, we asked them whether they had been previously infected with COVID-19 (“Yes, I had been and my COVID-19 test results were positive”, “I think I had been infected, but I didn’t do a medical COVID-19 test” or “No I don’t think so”). We only considered participants who answered the first option as being infected. Secondly, we asked them whether they had been placed in quarantine due to an actual or a potential infection with the COVID-19 (yes or no).

#### Risk Perception

We computed a risk perception indicator based on the estimated risk of infection and the perceived severity of COVID-19-related illness, following other recent studies using an indicator of risk perception (Schmitz et al., 2021; Wolff et al., 2019). We thus measured the estimated risk of infection with a 5-point Likert-type item: “*In your opinion, what is your risk of being infected with the coronavirus in the near future”*, and the perceived severity with a 5-point Likert-type item: “*In your opinion, if you were infected with the coronavirus, how serious would the consequences be?”*, both from very low (=1) to very high (=5).

### Procedure

We distributed a link to the online survey to all higher education students enrolled in the French speaking Belgian public educational system via their institutions and social media between February 22 to March 5, 2021. In Belgium, this time period corresponded to the second partial lockdown, which started in October 2020. Before they could fill in the survey, participants had to sign an informed consent form. In the first section, all participants (*N* = 20,048) provided sociodemographic information (e.g., age, gender) and completed questionnaires on general anxiety. Then, participants were randomly assigned to two out of four sections of the survey. The four sections were: (1) questionnaires on health anxiety, social support satisfaction and living alone; (2) questionnaires on compliance with the four sanitary behaviors; (3) questions on previous experiences with COVID-19 and academic motivation; and (4) questions on risk perception. Therefore, only half of the sample answered questions related to the four sanitary behaviors (N ranging from 10,035 to 10,107). Of these participants, one-third also answered the questions on section (1) with a N ranging from 3415 to 3441; another third answered the questions on section (3) with a N ranging from 3414 to 3441; and the last third answered questions on section (4), with a N ranging from 3201 to 3220. This explains the variation in the number of participants for each variable. Thus, each hierarchical regression was conducted on approximately 3000 participants.

This study was approved by the Ethics Committee of the Faculty of Psychology of the University of Liège.

### Data Analysis

We used IBM SPSS Statistics 27 to perform the data analyses. Because of the design of the study mentioned above, not all participants provided an answer to our predictor’ measures. Hence, we could not include all the predictors into the same regression model. To cope with this constraint, we developed three different regression models, which included a set of identical predictors across the three models (sociodemographic information and general anxiety), together with a subset of predictors that differed across the three models. The first model focused on mental health, including health anxiety, social support, and living alone. The second model included experiences with COVID-19 and academic motivation. The third model focused on risk perception. In order to manage missing values, the pairwise deletion method was used.

## Results

### Descriptive Statistics

Table 2 shows descriptive statistics. For the four sanitary behaviors, the mean scores showed a moderate level of compliance. The same pattern was observed for the predictors, except for risk perception, for which the average score was low. Most of the students lived with other individuals, such as family members or friends. Concerning COVID-19-related variables, half of the students have previously been in quarantine due to an actual or potential infection, and only 15% reported a previous infection with the COVID-19. The correlation matrices are available in supplementary materials (Appendix C).

**Table 2:** Descriptive statistics of dependent variables and predictors.


VARIABLES	N	MIN.	MAX.	MEAN	STD. DEVIATION	PERCENTAGES OF POSITIVE RESPONSE

Handwashing	10107	1	5	3.52	0.91	

Mask wearing	10084	1	5	3.15	1.17	

Physical distancing	10068	1	5	3.45	0.90	

Social distancing	10035	1	5	3.45	0.89	

General anxiety	20048	0	3	1.51	.62	

Health anxiety	10190	1	5	2.78	1.09	

Social support satisfaction	10181	1	5	3.05	.087	

Risk perception	9736	1	5	1.71	0.65	

Educational investment	10063	1	5	3.25	0.81	

Social network^1^	20048	1	2			90.8

Infection^2^	10047	1	2			14.8

Quarantine^3^	10047	1	2			55.1


*Notes*: ^1^ Percentages of people living with other individuals (e.g., parents, siblings, roommates, love partner). ^2^ Percentages of people who have been infected with the COVID-19. ^3^ Percentages of people who have been in quarantine due to the COVID-19.

### Hierarchical Regressions

Tables 3, 4 and 5 show the details of hierarchical regressions for each model respectively. The following sections summarize the results for each sanitary behavior.

**Table 3:** Summary of Hierarchical Regressions for Model 1 including Sociodemographic Variables and Mental Health in the Prediction of Sanitary Behaviors.


	HAND WASHING			MASK WEARING			PHYSICAL DISTANCING			SOCIAL DISTANCING		
			
	*β*	*CI (95)*	*F^2^*	*β*	*CI (95)*	*F^2^*	*β*	*CI (95)*	*F^2^*	*β*	*CI (95)*	*F^2^*

Gender	.121***	[.173; .297]	.020	.015	[–.045; .117]	.000	.066***	[.036; .190]	.000	.014	[–.035; .090]	.000

Age	.091***	[.028; .058]	.010	–.016	[–.029; .010]	.000	.087***	[.025; .056]	.010	.058***	[.011; .041]	.000

SES	–.024	[–.030; .004]	.000	.018	[.009; .034]	.000	.002	[–.016; .018]	.000	–.013	[–.024; .010]	.000

Anxiety	.101***	[.100; .198]	.010	–.007	[–.078; .051]	.000	.012	[–.033; .067]	.000	–.007	[–.060; .039]	.000

Health anxiety	.261***	[.188; .240]	.060	.303***	[.283; .352]	.087	.253***	[.179; .233]	.064	.267***	[.186; .239]	.075

Social support satisfaction	.034*	[.002; .069]	.000	–.001	[–.042; .045]	.000	–.002	[–.036; .032]	.000	–.029	[–.062; .004]	.000

Social network	.041***	[.032; .228]	.000	.045**	[.054; .310]	.000	.005	[–.085; .115]	.000	–.007	[–.118; .079]	.000

Model fit												

*R^2^*	.131			.094			.083			.077		

N	3441			3432			3428			3415		


*Note*: Gender is coded as a binary variable (1 = male, 2 = female). Participants with non-binary gender were removed from the analysis because they only represented 0.9% of the sample. Social network is coded as a binary variable (1 = living with others, 2 = living alone). * p < .05, ** p < .01, *** p < .001.

**Table 4:** Summary of Hierarchical Regressions in Model 2 including Sociodemographic variables, Experiences with COVID-19, and Motivation in Education in the Prediction of Sanitary Behaviors.


	HAND WASHING			MASK WEARING			PHYSICAL DISTANCING			SOCIAL DISTANCING		
			
	*β*	*CI (95)*	*F^2^*	*β*	*CI (95)*	*F^2^*	*β*	*CI (95)*	*F^2^*	*β*	*CI (95)*	*F^2^*

Gender	.120***	[.171; .299]	.020	.012	[–.055;.116]	.000	.041*	[.015; .147]	.000	.025	[–.018; .114]	.000

Age	.092***	[.028; .058]	.010	–.002	[–.021;.019]	.000	.086***	[.025; .056]	.010	.064***	[.014; .045]	.000

SES	–.036*	[–.035; –.002]	.000	.054**	[.014;.058]	.000	.031	[–.001; .033]	.000	.032	[–.001; .033]	.000

Anxiety	.198***	[.230; .327]	.031	.136***	[.184;.314]	.010	.152***	[.165; .265]	.020	.112***	[.107; .206]	.010

Contamination	–.086***	[–.241; –.102]	.010	–.143 ***	[–.465; –.280]	.020	–.138***	[–.350; –.207]	.010	–.144***	[–.358; –.215]	.020

Quarantine	–.042*	[–.141; –.014]	.000	–.029	[–.154; .015]	.000	–.042*	[–.143; –.013]	.000	.013	[–.041; .089]	.000

Motivation in education	.151***	[.131; .208]	.020	.144***	[.160;.262]	.020	.122***	[.099; .177]	.010	.101***	[.074; .152]	.010

Model fit												

*R^2^*	.089			.050			.057			.046		

N	3441			3431			3426			3414		


*Note*: Gender is coded as a binary variable (1 = male, 2 = female). Participants with non-binary gender were removed from the analysis because they only represented 0.9% of the sample. Social network is coded as a binary variable (1 = living with others, 2 = living alone). * p < .05, ** p < .01, *** p < .001.

**Table 5:** Summary of Hierarchical Regressions in Model 3 including Sociodemographic Variables and Risk Perception in the Prediction of Sanitary Behaviors.


	HANDWASHING			MASK WEARING			PHYSICAL DISTANCING			SOCIAL DISTANCING		
			
	*β*	*CI (95)*	*F^2^*	*β*	*CI (95)*	*F^2^*	*β*	*CI (95)*	*F^2^*	*β*	*CI (95)*	*F^2^*

Gender	.128***	[.176; .304]	.020	.020	[–.035;.134]	.000	.038*	[.006; .136]	.000	.039*	[.006; .134]	.000

Age	.066**	[.015; .045]	.000	–.047**	[–.047; –.007]	.000	.055**	[.009; .040]	.000	–.002	[–.016; .014]	.000

SES	–.003	[–.019; .016]	.000	.056**	[.015; .062]	.010	.021	[–.007; .029]	.000	.010	[–.013; .023]	.000

Anxiety	.127***	[.133; .232]	.010	.028	[–.013; .116]	.000	.047**	[.017; .118]	.000	.033	[–.003; .095]	.000

Risk perception	.155***	[.034; .052]	.020	.187***	[.054; 0.79]	.042	.109***	[.020; .039]	.020	.117***	[.022; .041]	.020

Model fit												

*R^2^*	.080			.043			.022			.019		

N	3220			3216			3209			3201		


*Note*: Gender is coded as a binary variable (1 = male, 2 = female). Participants with non-binary gender were removed from the analysis because they only represented 0.9% of the sample. Social network is coded as a binary variable (1 = living with others, 2 = living alone). * p < .05, ** p < .01, *** p < .001.

#### Handwashing

Being female, older, experiencing higher general anxiety and health anxiety, living alone, having higher academic motivation and higher risk perception were related to more compliance with handwashing. Having been infected or in quarantine were related to less compliance. An examination of effect sizes using Funder and Ozer’s guidelines (Funder & Ozer, 2019) revealed small effects of gender, age, anxiety, infection academic motivation, and risk perception, whereas health anxiety had a medium effect size. These predictors explained between 8% and 13% of the variance of handwashing.

#### Mask Wearing

Having a higher socioeconomic status, general anxiety, health anxiety, living alone, higher academic motivation and risk perception were related to more compliance with mask wearing. However, when health anxiety or risk perception were included in the models, general anxiety failed to reach the significance level. Moreover, being earlier in the academic curriculum and having been infected were related to less compliance. Analyses revealed small effect sizes for socioeconomic status, anxiety, past infection, academic motivation, but medium effects for health anxiety and risk perception. These predictors explained between 4.3% and 9.4% of the variance of mask wearing.

#### Physical Distancing

Being female, more advanced in the curriculum, displaying higher general and health anxiety, having higher academic motivation, and risk perception were associated with more compliance with physical distancing. Having been infected or in quarantine were related to less compliance. Regarding the effect sizes, age, anxiety, past infection, academic motivation and risk perception had small effect sizes, whereas health anxiety had a medium effect size. These predictors explained between 2% and 8% of the variance of physical distancing.

#### Social distancing

Being older, scoring higher on general and health anxiety, having higher academic motivation, and reporting higher risk perception were related to more compliance with social distancing. However, when health anxiety or risk perception were added to the models, the influence of general anxiety was not significant anymore. Social support satisfaction and having been infected with COVID-19 were related to less compliance. Analysis of effect sizes revealed small effects of anxiety, infection, academic motivation, and risk perception, whereas a medium effect size was found for health anxiety. These predictors explained between 2% and 7.7% of the variance of social distancing.

## Discussion

This cross-sectional study investigated factors predicting adherence to sanitary behaviors in a population of higher education students aged between 18 and 25. We investigated four sanitary behaviors considered as fundamental to limit the propagation of COVID-19 (World Health Organization, 2020): handwashing, mask wearing, physical distancing, and social distancing.

Results showed that higher general anxiety, health anxiety, previous experiences with COVID-19, higher academic motivation, and higher risk perception predicted these four preventive behaviors. Although previous studies have also investigated tertiary-level students’ adherence to sanitary behaviors (Cohen et al., 2020, 2021; DeJonckheere et al., 2021; Park et al., 2020; Varol et al., 2021), the present study drew on data from more than 3000 participants for each condition, whereas the sample size in earlier studies ranged from only 255 (e.g., Varol et al., 2021) to 1015 (e.g., Park et al., 2020) participants.

First, descriptive results showed that sanitary behaviors were moderately adopted, with means ranging from 3.15 to 3.52 on a 5-points scale. These results, especially for social distancing, were a bit lower than in other Belgian sample of college students (e.g., Wismans et al., 2020). First, this moderate adherence might be explained by a lower motivation to comply after one year in the pandemic. A second explanation for this moderate compliance was the low rate of infection in the sample, as only 15% of the students reported previous infection with the COVID-19 since the beginning of the pandemic.

Moreover, regression analysis showed that being older had a positive impact on adherence to handwashing, physical distancing, and social distancing. Firstly, this age effect could be interpreted as a developmental effect. Indeed, younger college students were less prone to comply because of their less mature brains regarding risk-taking. Adolescence and early adulthood is a sensitive period of brain development (Fuhrmann et al., 2015). The brain reaches its full maturation around 25 years old, with the prefrontal cortex having the latest development (Arain et al., 2013). Particularly, frontal regions are involved in self-regulation abilities that include impulse control and risk-taking (Miyake & Friedman, 2012). Therefore, young adults are more prone to engaged in risky behaviors. Moreover, a recent study found that ambiguous conditions increase the propensity to take risks among young adults (Ogilvie et al., 2020). At the time of data collection, infection rates associated to the COVID-19 raised again after a period of lull (Sciensano, 2021a, 2021b), leading to a time of uncertainty for college students. This ambiguous situation might explain why younger students in college tended not to comply with COVID-19-related sanitary behaviors. However, it is worthwhile restating that we restricted the age range to 18 and 25 years old, and age in our sample was closely related to the students’ year of study (*r* = .65). Then, this effect of age on compliance can also be interpreted as an effect of the year of study on students’ compliance. Indeed, being later in the academic curriculum was associated with a more developed critical thinking, which is related to health protective behaviors during the COVID-19, especially for handwashing and social distancing (Čavojová et al., 2022; Swami & Barron, 2021). Finally, in Belgium, at the time of our study, preventive measures implemented regarding tertiary-level students were dependent on the advancement in study years. Specific measures allowed students in their first year of bachelor to continue face-to-face courses in small groups on campus. By contrast, students studying for master-level degrees could only attend online classes, preventing them from participating in a great number of social contacts and outdoor activities. This difference between bachelor and master-level students may be reflected in the data, and be another explanation of the age effect found.

Then, a positive relationship between academic motivation and compliance with preventive behaviors was found. That is, college students who were more invested in their studies adopted health guidelines to a greater extent than students who were less invested. This significant association might be explained by the association between academic motivation and level of conscientiousness (Hazrati-Viari et al., 2012), which has already been highlighted as a predictor of adherence to COVID-19-related behaviors (Blagov, 2021; Krupić et al., 2021; Moore et al., 2022; Otterbring & Festila, 2022). Conscientiousness is also a personality trait involved in academic motivation among college students before and during times of COVID-19 (Audet et al., 2021; Komarraju et al., 2009; Önder et al., 2014). Therefore, it is possible that conscientiousness played a role as a third variable in explaining the association between higher motivation in education and adherence to sanitary behaviors. An in-depth analysis of the relationships between these three variables would help to understand our results. Future studies should consider investigating the impact of personality traits such as conscientiousness in predictive models of COVID-19-related behaviors.

In addition to these substantive issues, an original contribution was the use of distinct indices to measure the four sanitary behaviors, with each including a set of contexts that varies in its ease of application. These four indices allowed us to investigate the different processes and key variables of their respective preventive behaviors. These scales were already used in others predictive studies related to COVID-19 behaviors (Wollast et al., 2021). Despite the medium size correlations between the sanitary behaviors, ranging from .38 to .67 (Table C1 in supplementary materials), we identified a set of very similar predictors across the behaviors, which were close to other results on the Belgian population (Schmitz et al., 2022).

Among them, mental health variables and risk perception seemed to play a key role in adherence. The role of general anxiety for compliance was consistent with recent research on handwashing (Solomou & Constantinidou, 2020). Our results extended the effect of anxiety on wearing the facial mask, maintaining a physical distance, and limiting social contacts. Although general anxiety significantly predicted these four behaviors, health anxiety was a predictor of adherence above and beyond general anxiety, with a medium effect size for the four behaviors. For mask wearing and social distancing, general anxiety even failed to reach significance when health anxiety was included in the model. Thus, being specifically anxious about health seems to be a reliable predictor of sanitary behaviors in the context of the COVID-19 pandemic. This finding was consistent with the observation that adherence to COVID-19 preventive guidelines was associated with higher fear of COVID-19 (Harper et al., 2021; Parlapani et al., 2020). Even if higher health anxiety was associated with more compliance with sanitary behaviors, focusing on this domain is not a recommended strategy. Indeed, increasing health anxiety could intensify psychological distress and maladaptive compliance with sanitary behaviors (Sauer et al., 2020).

Results also revealed that risk perception was another predictor of higher compliance with COVID-19-related behaviors, especially for mask wearing. This finding followed previous studies among college students (Batra et al., 2021; Borges & Byrne, 2022; Rayani et al., 2021). Risk perception in our sample was lower than other studies (Ding et al., 2020; Patil et al., 2021), but it can be explained by the Belgian situation at the time of data collection, as staying at home was mandatory for students. Therefore, students were less at risk to be infected with the coronavirus. It is also relevant to note that male and female students differed from each other regarding their risk perception level. Indeed, female students were more compliant than their male counterparts, and perceived also more risk of infection for the COVID-19. This gender gap was consistent with previous studies suggesting that men usually perceived a lower risk of infection for COVID-19 (Lewis & Duch, 2021; Rana et al., 2021; Rodriguez-Besteiro et al., 2021). Taken together, these results suggested the need to target their risk perception, especially among male students (Lewis & Duch, 2021). Tailored messages for students in higher education that emphasize the risk they pose to themselves and their close relatives in neglecting sanitary behaviors can increase their risk perception. The use of visual materials to support these messages is highly recommended (Motivation barometer, 2021e, 2021f).

Simultaneously, as other research (Dryhurst et al., 2020), we found that individuals who had been infected with COVID-19 were less likely to adopt preventive behaviors, suggesting that having been infected gives the illusion of protection against the virus. Consequently, it is worthwhile addressing these inaccurate beliefs in the young population. In their longitudinal study on vaccination, Schmitz and colleagues (Schmitz et al., 2021) showed the decisive role of risk perception and its relation to adaptive adherence. Future preventive interventions and COVID-19-related communications should focus on increasing the level of risk perception among the college student population rather than playing on their worries or anxieties. Increasing risk perception, especially among students with high health anxiety, seems to be the safest way to preserve their mental health. Indeed, it could allow students to escape maladaptive health anxiety by focusing on more adaptive adherence to preventive behaviors. This is especially accurate among women because of their higher propensity to use health anxiety as a drive for preventive behaviors (Alsharawy et al., 2021). Hence, communication should prioritize showcasing the severity of the illness among the young population (Schmitz et al., 2021) to increase the risk perception and the uptake of preventive sanitary behaviors.

It is important to acknowledge that the explained variance of our models remained quite low. This was due to the fact that some central factors were not considered, such as those featured in the socio-cognitive models of behavior change (e.g., Theory of Planned Behavior). These models were thought to be key determinants of sanitary behaviors in general, and in this specific time of pandemic (Bigot et al., 2021; Chon & Kim, 2022; Farias & Pilati, 2022; Gibson et al., 2021; Wismans et al., 2020; Wollast et al., 2021). Indeed, research found that positive attitudes towards the behavior, perceived behavioral control, and intention predicted more handwashing (Bigot et al., 2021; Schmitz et al., 2022; Wollast et al., 2021), whereas the intention to wear a mask was associated with subjective norms (Zahed et al., 2021) and attitude (Irfan et al., 2021). Regarding social distancing, findings were not so conclusive across studies. Bigot and colleagues (2021) found that intention, subjective norms, and attitude, but not perceived behavior control, increased social distancing, whereas perceived behavioral control, subjective norms and attitude were found to play a positive role in the intention to adopt social distancing (Farias & Pilati, 2022; Gibson et al., 2021). These relevant predictors were not included in the present study as the initial goal was to provide an overall description of mental health and education difficulties faced by students during the second partial lockdown in Belgium (Schmits et al., 2021). Thus, the initial goal of this study was exploratory.

One further limitation of the study was that the cross-sectional design did not allow for causal inferences. Thus, futures studies should implement longitudinal designs to investigate the stability of the predictors we found over time, including variations of the pandemic severity (Schumpe et al., 2022; Stroebe et al., 2021; Wollast, Schmitz, et al., 2022). Also, our sample included an overrepresentation of women. Finally, although the sample was similar to the French-speaking Belgian student population (Académie de Recherche et de l’Enseignement Supérieur, 2016), students enrolled in universities were over-represented, and those in colleges were under-represented.

These characteristics notwithstanding, the results of our study dovetailed remarkably well with past studies on determinants of sanitary behaviors during pandemics (Bish & Michie, 2010; Wheaton et al., 2012) and should be used to help guide policy-making in times of COVID-19.

## Data Accessibility Statement

Data are available on OSF at this link: https://osf.io/kwzyn/?view_only=2c299541d38a483c82c0dd9e1b12a032.
